# Periostin in allergic rhinitis: from pathogenic mediator to predictive biomarker and therapeutic target

**DOI:** 10.3389/fphar.2026.1801272

**Published:** 2026-06-18

**Authors:** Han Wang, Qihang Zhang, Miao Hu, Junhao Shao, Guangke Wang

**Affiliations:** 1 Department of Otorhinolaryngology and Head and Neck Surgery, Zhengzhou University People’s Hospital, Zhengzhou, Henan, China; 2 Department of Otorhinolaryngology and Head and Neck Surgery, People’s Hospital of Henan University, Zhengzhou, Henan, China; 3 Department of Otorhinolaryngology and Head and Neck Surgery, Henan Provincial People’s Hospital, Zhengzhou, Henan, China

**Keywords:** allergic rhinitis, biomarker, periostin, precision medicine, tissue remodeling, type 2 inflammation

## Abstract

Allergic rhinitis (AR) is a common disease around the world, mediated by type 2 immune responses, and some patients don’t respond well to current standard treatments. This shows AR isn’t a single type of disease but a complex syndrome caused by different internal body mechanisms. Periostin is a key matrix protein sitting downstream of the interleukin-4 (IL-4) and interleukin-13 (IL-13) pathways, connecting type 2 immune responses and nasal mucosal tissue remodeling. This article explains periostin’s many roles in AR: it breaks down the epithelial barrier, makes eosinophils gather, and causes fibrosis, and these things form a never-ending “inflammation-repair” vicious cycle. In clinical use, periostin levels in different biological samples matter—they relate to how severe the disease is and show how patients respond to glucocorticoids and biologics. Targeting periostin directly might offer new ways to help with hard-to-treat tissue remodeling. Based on these findings, we suggest a way to classify AR using periostin levels, which aims to guide personalized treatment in the future and help AR diagnosis and therapy become true precision medicine.

## 1 Introduction

**FIGURE 1:**
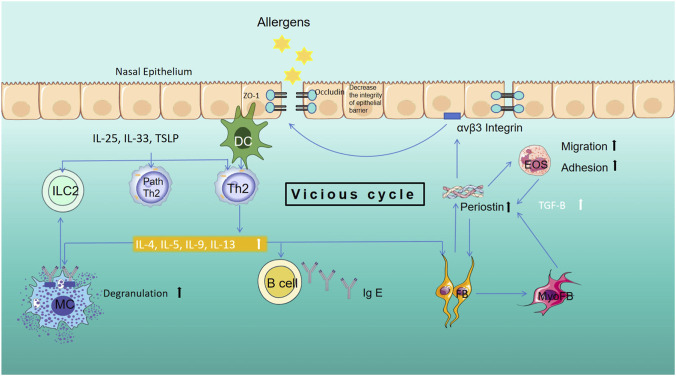
Allergen exposure triggers the release of type 2 cytokines (particularly IL-4/IL-13). IL-4 and IL-13 can induce the expression of periostin in fibroblasts. Periostin promotes inflammation and tissue remodeling through mechanisms such as disrupting the epithelial barrier, recruiting and activating eosinophils (Eos), activating fibroblasts (FB), and inducing their transformation into myofibroblasts (MyoFB). Activated Eos and MyoFB further produce mediators such as TGF-β, which in turn enhance periostin expression, forming a self-perpetuating positive feedback loop.

Allergic rhinitis affects hundreds of millions of patients worldwide, lowering their quality of life and bringing a big socioeconomic burden (Cingi et al., 2019; Hong et al., 2020). Nasal corticosteroids and antihistamines are common treatments that work well for many (Brożek et al., 2017), but patients often face problems: responses to treatment vary a lot, symptoms come back after stopping treatment, and chronic disease leads to nasal mucosal tissue remodeling which causes hard-to-treat nasal obstruction (examples include basement membrane thickening and fibrosis). These differences show AR isn’t one disease but a syndrome triggered by different internal mechanisms—doctors call this “endotypes”. Th2 cytokines play a major role, and IL-4 and IL-13 are especially important: they drive type 2 inflammation and are a common core pathological pathway for AR (Gandhi et al., 2017) (Figure 1).

IL-4 and IL-13 are short-lived signaling molecules that need stable downstream effectors to have long-term pathological effects, and periostin is a key intermediate molecule in this process (Liu et al., 2025). IL-4 and IL-13 induce periostin through the STAT6 pathway, and periostin turns immune signals into lasting changes in the stromal microenvironment. Periostin has different roles: it affects tissue development and influences the progression of many diseases, including allergic rhinitis, asthma, dental diseases, cardiac diseases, and osteoarthrosis (Yang et al., 2022). In diseases like asthma, periostin’s role is proven—it’s a critical mediator and a predictive biomarker (Woodruff et al., 2009). Its value in allergic rhinitis is becoming clearer, and this article aims to explain periostin’s main roles in AR’s pathogenesis, evaluate its use as a clinical biomarker, review the current state of targeted therapies, and explore precision diagnosis and treatment paths guided by this molecule.

## 2 Biological properties of periostin

Periostin is encoded by the Postn gene and was first identified in 1993 when researchers found it in a mouse osteoblastic cell line (Takeshita et al., 1993). It has a molecular weight of 90 kDa, belongs to the fasciclin-containing family, and is part of the extracellular matrix but not a structural component (Takayama et al., 2006). It binds to integrin receptors on cell surfaces—examples are αvβ3 and αvβ5—and this binding regulates the behavior of various cells, including epithelial cells and fibroblasts (Izuhara et al., 2019).

Allergic rhinitis starts with allergen exposure, which triggers nasal mucosal epithelial cells to release alarm molecules like TSLP and IL-33 (AlBloushi and Al-Ahmad, 2024). These molecules activate downstream Th2 cells and type 2 innate lymphocytes (ILC2s), and the activated cells produce high levels of IL-4 and IL-13. IL-4 and IL-13 are key signals inducing periostin expression: they activate the JAK-STAT6 pathway, which promotes the transcription of the Postn gene (Izuhara et al., 2014; Shen et al., 2018). Fibroblasts and epithelial cells in the nasal mucosa are the main sources of periostin (Takayama et al., 2006; Mitamura et al., 2018a). Clinical studies have confirmed a link—periostin levels correlate positively with IL-4 and IL-13 concentrations in nasal lavage fluid from AR patients—providing direct evidence that periostin is a major effector molecule downstream of type 2 inflammation (Ishida et al., 2012).

## 3 Periostin drives a self-sustaining vicious cycle

Periostin doesn’t just react to inflammation passively; it actively takes part in AR’s pathological process and makes the process worse (Zhu et al., 2025). Its main function is to create a self-reinforcing “inflammation-tissue remodeling” vicious cycle.

### 3.1 Amplification of type 2 inflammation

Periostin makes early inflammatory responses worse directly through multiple mechanisms. It binds to integrin receptors like αvβ3 to disrupt the epithelial barrier (Chung et al., 2020)—this binding starts intracellular downstream signaling cascades that cause abnormal cell signal transduction, impairing the functionality of key tight junction proteins like occludin-1 and occludin (Nur Husna et al., 2021). The nasal mucosal epithelium’s barrier integrity is compromised both functionally and structurally, letting allergens invade the body more easily.

Periostin recruits and activates eosinophils as an efficient eosinophil chemokine (Nur Husna et al., 2021; WITHDRAWN, 2024). Animal experiments have clear results: mice with the periostin gene knocked out show less eosinophil infiltration into the nasal mucosa induced by allergens (Hur et al., 2012). Periostin also helps eosinophils survive longer and improves their degranulation capacity through the integrin signaling pathway (Ohta et al., 2014).

### 3.2 Promotion of tissue remodeling

This effect is key to making allergic rhinitis chronic and hard to treat. When the body is exposed to high levels of IL-4/IL-13 for a long time, it leads to sustained high expression of periostin, and then a series of tissue changes occur.

Periostin directly promotes fibrosis by inducing fibroblasts to synthesize large amounts of type I and type III collagen, triggering subepithelial fibrosis (Yang et al., 2014; Mitamura et al., 2018b). In nasal mucosa biopsy specimens from AR patients, periostin is often seen colocalized with α-smooth muscle actin-positive activated fibroblasts (Conway et al., 2014).

Periostin can induce glandular hyperplasia and goblet cell metaplasia (O'Dwyer and Moore, 2017), which are characteristic of chronic allergic rhinitis.

Periostin works with remodeling-promoting pathways through synergistic effects. Periostin and transforming growth factor-β (TGF-β) are the core signaling hub driving tissue remodeling (Nishizawa et al., 2012), forming a bidirectional positive feedback loop. Using the integrin-Fak-SRC signaling pathway, periostin directly induces fibroblasts to synthesize TGF-β and upregulates the expression of TGF-β type II receptors on cell surfaces, making cells more sensitive to low concentrations of TGF-β in the microenvironment (this is called the “sensitization” effect). On the other hand, activating the TGF-β/Smad signaling pathway also increases periostin expression (Conway et al., 2014; Snider et al., 2009). This interactive amplification mechanism is the molecular basis for inflammatory responses to turn into irreversible tissue remodeling.

### 3.3 Positive feedback loop

Inflammatory cells like eosinophils are recruited to the lesion site, and these cells produce more mediators such as TGF-β and IL-4/IL-13. These mediators stimulate interstitial cells to express more periostin, creating a self-sustaining vicious cycle: “IL-4/13 - periostin - inflammation and remodeling - release of more inflammatory mediators (like TGF-β) - continuous periostin production” (Figure 1).

## 4 Periostin and clinical symptoms of AR

The molecular mechanisms driven by periostin show up directly as the clinical signs and symptoms of AR patients.

Although a direct link between sneezing, nasal itching and periostin has not been formally established (Chung et al., 2020; Nur Husna et al., 2021), periostin is known to disrupt the epithelial barrier and promote eosinophilic inflammation (WITHDRAWN, 2024; Hur et al., 2012; Ohta et al., 2014), suggesting a plausible connection to these clinical manifestations of AR.

Rhinorrhea symptoms are closely linked to periostin. Periostin induces glandular hyperplasia and goblet cell metaplasia, a pathological change that enhances the nasal mucosa’s secretory capacity. At the same time, it promotes eosinophil aggregation and activation, indirectly stimulating the release of mediators that make glands secrete more, and finally leading to more nasal discharge in patients.

Nasal obstruction is the most clinically significant symptom caused by periostin. Periostin drives subepithelial fibrosis formation and promotes extracellular matrix deposition, leading to persistent structural changes in the nasal mucosa. This type of nasal obstruction is different from the transient mucosal swelling from vascular congestion—it’s refractory nasal obstruction that doesn’t respond well to conventional decongestants and glucocorticoids.

Periostin promotes nasal hyperresponsiveness through multiple mechanisms. On one hand, it induces eosinophilic inflammation and releases neurotrophic factors, sensitizing sensory nerve endings. On the other hand, structural changes in the nasal mucosa affect the airway’s biomechanical properties, making the airway more responsive to nonspecific stimuli like temperature changes and irritant gases.

## 5 Periostin as a clinical biomarker in AR

Periostin has specific expression and is relatively stable, making it a promising biomarker, but its value needs to be analyzed with sample types and clinical scenarios.

### 5.1 Sample-specific considerations and published levels

Table 1 summarizes published periostin levels, including those from different biological matrices, measurement methods, and patient populations.

**TABLE 1:** Published periostin levels in allergic rhinitis.

Sample matrix	Reported levels	Measurement method	Population	Key findings	Reference
Serum	No significant difference	ELISA	AR children (Korea)	Levels affected by age and growth; local samples may be more discriminative in pediatrics	Kim et al. (2017)
Serum	26.18 (20.85–49.21) ng/mL (AR) vs. 18.95 (17.43–23.26) ng/mL (control)	ELISA	AR patients (adults, China)	Elevated in AR vs. controls; correlated with FeNO, FnNO, and ocular tearing score; AUC 0.773 for AR diagnosis	Zhu et al. (2025)
Nasal Secretions	30.21 ± 17.60 ng/mL (AR) vs. 11.09 ± 20.60 ng/mL (control)	ELISA	AR patients (adults, China)	Significantly higher in AR than controls; positively correlated with IL-4 (r = 0.628) and IL-13 (r = 0.860)	Gu et al. (2024)
Nasal Lavage Fluid	215.2 ± 87.7 pg/mL	ELISA	AR patients (adults, China)	Correlated with IL-5; decreased after posterior nasal neurectomy	Wang et al. (2020)
Nasal Mucosa Tissue	Strong IHC staining	Immunohistochemistry	AR patients	Co-localized with α-SMA + fibroblasts; associated with remodeling	Conway et al. (2014)

Abbreviations: ELISA, enzyme-linked immunosorbent assay; IHC, immunohistochemistry; AR, allergic rhinitis.

### 5.2 Core clinical application scenarios

Identification of patients with remodeling predisposition: High periostin content, particularly localized levels, indicates a higher likelihood of developing chronic and refractory AR subtypes.

#### 5.2.1 Predicting therapeutic response

Glucocorticoids: Evidence is inconsistent with conflicting conclusions from different studies. Some studies found periostin levels decrease in patients who respond to treatment (Matsumoto, 2014), while other studies suggest high periostin levels may relate to poor efficacy of nasal corticosteroids (Yang et al., 2020) or indicate existing tissue remodeling makes the tissue insensitive to corticosteroids (Tai et al., 2005).

Biologics: This is the most transformative use. Asthma studies have shown that high baseline serum periostin levels can identify patients who respond better to IL-13 monoclonal antibodies like lebrikizumab (Scheerens et al., 2014; Corren et al., 2011). For allergic rhinitis, dupilumab targeting IL-4Rα has shown efficacy (Castro et al., 2018), and as a downstream molecule of dupilumab, periostin has potential as a companion diagnostic biomarker to predict therapeutic efficacy—though more AR-specific data is needed for validation.

Allergen-Specific Immunotherapy: Besides biologics, serum periostin has predictive value for response to this therapy. Hoshino et al. did relevant studies and found higher baseline serum periostin levels in AR patients were associated with better clinical responses to sublingual immunotherapy for house dust mites (Hoshino et al., 2021). These findings show periostin’s practical use in identifying patients suitable for immunotherapy and helping choose more individualized treatment strategies.

### 5.3 Standardization challenges

There are several challenges: standardizing detection methods and sampling procedures is a current bottleneck, reference value ranges haven’t been established for different age groups and populations, and short-term factors like acute infections can cause numerical fluctuations. Future multicenter, prospective studies are needed to validate periostin’s predictive value.

### 5.4 Evidence contradictions and interpretation

Most studies support the conclusion that serum periostin expression is elevated in AR patients, but there are conflicting research findings. Selmonaj Rama’s team did relevant studies and found no significant differences in serum periostin levels between AR patients, non-allergic rhinitis patients, AR patients with lower respiratory symptoms, and healthy controls (Selmonaj Rama et al., 2024). Multiple factors may cause these different results.

Population characteristics like age, ethnicity, and comorbidities can influence outcomes as they may affect baseline periostin levels. Serum periostin levels change with age and bone metabolism, making test results harder to interpret—especially for pediatric or diverse adult populations (Kim et al., 2017). Methodological differences in studies, such as variations in sample processing, testing platforms, and cutoff value settings, can also lead to inconsistent results and reduce comparability between studies. Additionally, periostin is mainly a marker of tissue remodeling rather than acute inflammation, so without significant tissue remodeling, its levels can’t tell AR apart from other rhinitis subtypes.

These findings point to future research directions: there’s an urgent need to establish standardized detection protocols and conduct larger-scale prospective cohort studies to clarify periostin’s practical value in AR diagnosis and endotyping.

## 6 Targeting the periostin pathway

### 6.1 Upstream inhibition

IL-4/IL-13 pathway inhibitors represented by dupilumab have been successfully used in clinical therapy. These drugs’ core mechanism of action is disrupting upstream signal transduction to inhibit downstream periostin production (Corren et al., 2011). Clinical trials have clear results: serum periostin levels are significantly reduced in patients who respond to treatment (Hamilton et al., 2021). Though these studies didn’t directly measure periostin as a primary endpoint, the therapeutic mechanism of IL-4/IL-13 signal blockade strongly suggests it can suppress downstream periostin expression. This indirect evidence highlights the periostin pathway’s significance in biologic therapies for type 2 inflammation, and dynamic changes in periostin expression levels can be used as biomarkers to monitor therapeutic responses.

Dupilumab has shown good efficacy in various diseases, including asthma, atopic dermatitis, and chronic rhinosinusitis with nasal polyps. The therapeutic effect comes partly from its dual action of inhibiting inflammatory responses and stopping tissue remodeling. Phase III clinical trials for chronic rhinosinusitis with nasal polyps—like the NCT02898454 trial—show dupilumab is much more effective than placebo: it improves 22 sinusitis symptom scores, enhances patients’ quality of life scores, and reduces levels of inflammatory markers like peripheral blood eosinophils (Fujieda et al., 2021).

### 6.2 Direct periostin neutralization

Targeting periostin itself directly or its integrin receptor binding site offers more precise intervention, but this approach is still in the exploratory phase.

Studies on monoclonal antibody applications have been done in mouse asthma models, where anti-periostin neutralizing antibodies showed therapeutic effects by reducing airway hyperresponsiveness, alleviating inflammatory responses, and inhibiting mucus secretion (Bentley et al., 2014). But mouse model findings need careful interpretation—observed therapeutic effects may come from nonspecific immunomodulation and may not fully apply to human nasal mucosal tissues. Further validation in human tissues is essential before clinical translation, and target specificity also needs confirmation.

Small molecule inhibitor development focuses on disrupting the binding between periostin and integrins. No studies have reported using periostin-targeting inhibitors for AR treatment, but preclinical research on other fibrotic diseases has explored inhibitors that can block periostin-integrin interactions. This therapeutic strategy is still in early development and hasn’t entered clinical evaluation for AR.

Directly targeting periostin has potential advantages but also faces unique challenges. The potential advantages lie in intervention precision—it may allow more targeted modulation of started tissue remodeling processes—but two core challenges need to be addressed.

Safety considerations are the primary challenge: periostin plays important physiological roles in the body, such as in bone remodeling and cardiac repair (Kanemitsu et al., 2013; Shiraishi et al., 2012; Snider et al., 2008; Oku et al., 2008), so systemically inhibiting periostin expression may pose safety risks. Developing function-specific antibodies—like those that only block binding to pathological integrin subtypes—or exploring tissue-targeted delivery systems may help expand the drug’s therapeutic window.

Delivery challenges are another major core issue: local administration for AR faces inherent limitations for macromolecular drugs, such as poor mucosal permeability, easy enzymatic inactivation, and rapid clearance (Ulusoy et al., 2022). Emerging technologies like biomimetic nanocarriers, elastic liposomes, and cell-penetrating peptide fusion techniques provide solutions—they can prolong drug retention time in the body, enhance subepithelial penetration efficiency, and may bring breakthroughs in AR’s local treatment (Jacob et al., 2020; Hussain et al., 2017; Kanazawa et al., 2013). So, directly targeting periostin is theoretically an effective method to disrupt the “inflammation-repair” cycle, but its successful translation depends on two breakthroughs: highly selective drug design and effective local delivery technologies.

## 7 A periostin-based endotyping framework for AR

Based on the above evidence, we propose a precision typing framework that integrates periostin into clinical decision-making. Table 2 summarizes the framework’s core components, which are the endotype core characteristics of AR based on periostin.

**TABLE 2:** Periostin-based endotyping framework for allergic rhinitis.

Feature	Periostin-high subtype	Periostin-low subtype
Periostin Levels	Persistently elevated in serum and/or nasal samples (Jia et al., 2012)	Normal range or slightly elevated
Inflammatory Profile	Marked type 2/eosinophilic inflammation (Kanazawa et al., 2013)	May involve non-type 2 pathways (Th1/Th17) (Ricciardolo et al., 2021) or neurogenic inflammation
Tissue Remodeling	Definitive evidence present (biopsy or imaging)	No significant evidence
Clinical Phenotype	Moderate-to-severe, persistent symptoms; often with comorbid asthma; possible poor response to conventional nasal corticosteroids (Ninomiya et al., 2018; De Schryver et al., 2017; Jonstam et al., 2017; Maxfield et al., 2018; Xu et al., 2018)	Symptom severity varies; remodeling-related symptoms (e.g., fixed obstruction) rare
Treatment Strategy	Primary candidates for targeted biologics (anti-IL-4Rα, anti-IL-13) (Brightling et al., 2015); assess baseline periostin in inadequately controlled patients	Optimize conventional pharmacotherapy; investigate alternative inflammatory pathways; avoid indiscriminate biologic use
Monitoring	Periostin dynamics may guide treatment adjustment	Periostin not primary monitoring tool

### 7.1 Validation and implementation

This classification framework’s practical application needs validation through prospective studies. Table 3 outlines relevant research’s key findings, identifying periostin as a critical step and research objective to establish it as a clinically applicable biomarker for stratified treatment of AR.

**TABLE 3:** Validation framework for periostin-based stratified treatment in AR.

Validation phase	Key task	Specific content	Objective
1. Establishment of Detection Standards	Determine optimal test samples and unified thresholds	- Compare the sensitivity and specificity of local samples (nasal secretions, tissues) and serum samples- Develop promotable detection methods and cutoff values	Provide a standardized detection protocol for periostin as a biomarker
2. Construction of Prediction Model	Integrate multi-dimensional indicators to improve the accuracy of classification diagnosis	- Combine periostin, blood eosinophil count and specific IgE- Develop machine learning or statistical models	Enhance the precision of patient subtype classification and identify populations responsive to targeted therapy
3. Design of Prospective Trials	Verify the effectiveness of stratified therapy	- Group patients by preset periostin levels (high/low)- Compare efficacy differences between biologics and standard therapy (e.g., symptom scores, inflammatory markers)- Conduct long-term follow-up to evaluate treatment response rate	Provide evidence-based medical evidence to confirm the clinical value of periostin as a navigation tool for stratified therapy
4. Clinical Application Objective	Guide precise treatment strategies	- Select individualized treatment plans based on periostin levels (e.g., anti-IL-4/13 biologics, immunomodulators)- Dynamically monitor changes in periostin during treatment	Optimize the prognosis of AR patients, reduce ineffective treatment, and lower medical costs

Current prospective study evidence is limited—it can’t confirm that AR patients with high periostin expression have better therapeutic outcomes with biologic therapy. This is a critical gap in AR research that targeted clinical trials are urgently needed to fill. This framework’s core clinical value is using periostin as a stratification tool to tailor the best treatment plans for individual patients. The structured validation methodology in Table 3 provides a clear roadmap for future research to fill this knowledge gap and promote translating periostin-based endotyping into clinical practice. Periostin has stable expression levels and relative specificity, making it a very promising biomarker, but its practical clinical value needs comprehensive evaluation considering sample types and real clinical scenarios.

## 8 Future perspectives

Future research on periostin will focus on multiple key directions. Standardizing detection methods is the primary focus, with critical steps including revising tissue sampling protocols and establishing standardized techniques for periostin and POSTN messenger RNA detection to build a reliable research platform. Conducting prospective cohort studies is another essential direction to clarify periostin’s practical predictive value—especially for evaluating therapeutic efficacy in AR patients undergoing biologic therapies and allergen-specific immunotherapy.

Breakthroughs in drug development are the core direction: research on inhibitors that directly act on periostin will be accelerated, with priority given to developing nasal local formulations, and early safety assessments and evaluations of tissue remodeling reversal effects will be conducted. The integrated diagnosis and treatment model is the developmental direction—it incorporates periostin-based endotyping into the holistic management framework of “one airway, one disease” to achieve systematic and individualized treatment for AR and its common comorbidities.

## 9 Conclusion

Periostin plays a key role in AR: it’s the core pathogenic mechanism, a key driver of tissue remodeling, a precise measurement standard, and a novel therapeutic target. It clearly explains how type 2 inflammation progresses from a short-term immune response to long-term structural lesions. In summary, understanding periostin well is leading AR into a new era of precision medicine driven by intrinsic mechanisms. Identifying AR patient subgroups with high periostin expression and using targeted therapeutic approaches for them may help shift treatment from “symptom control” to “disease modification”.

## Research methods

**FIGURE 2:**
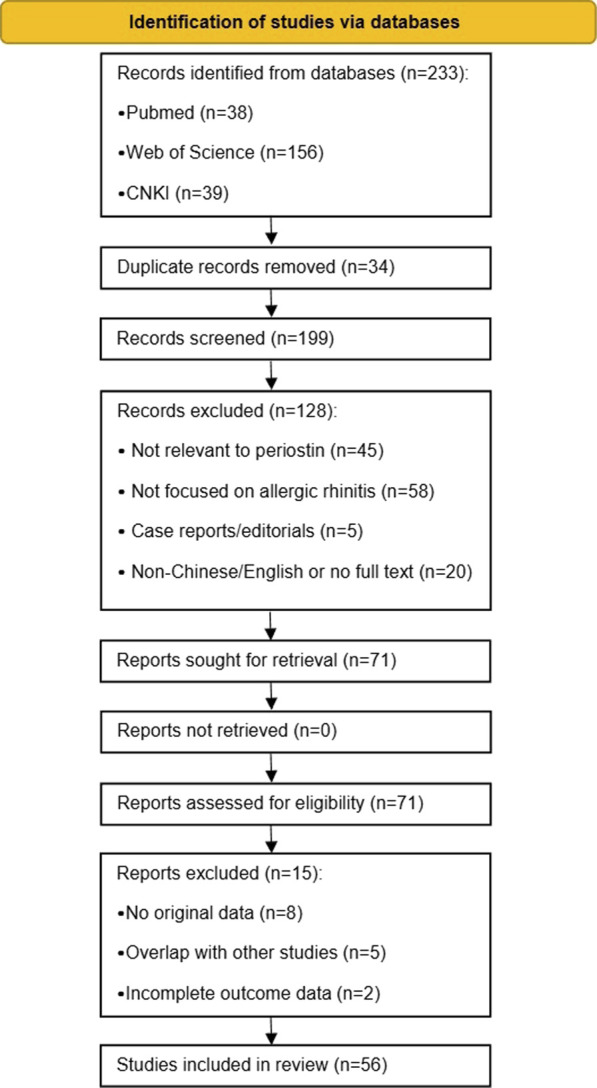
PRISMA 2020 flow diagram. Databases: PubMed, Web of Science, CNKI (search date: January 2026). 233 records identified → 34 duplicates removed → 199 screened → 128 excluded → 71 full-text assessed → 15 excluded → 56 studies included.

This study conducted a systematic review following the PRISMA guidelines. We searched PubMed, Web of Science, and CNKI from inception to January 2026 using keywords including “periostin”, “allergic rhinitis”, “POSTN”, “biomarkers”, “type 2 inflammation”, and “tissue remodeling”. We included original research, clinical studies, and reviews focused on periostin in allergic rhinitis, and excluded non-Chinese/English literature, case reports, and studies without full text. Two researchers independently screened titles, abstracts, and full texts. The screening process is documented in the PRISMA flow diagram (Figure 2), and the PRISMA 2020 checklist is provided as supplementary material. Data were extracted using a standardized form, and findings were synthesized narratively.

## Data Availability

The original contributions presented in the study are included in the article/Supplementary Material, further inquiries can be directed to the corresponding author.
