# Supplementary material for: Periostin in allergic rhinitis: from pathogenic mediator to predictive biomarker and therapeutic target
Source: Front Pharmacol. 2026 Jun 18;17:1801272. doi: 10.3389/fphar.2026.1801272 (PMC13322882; doi:10.3389/fphar.2026.1801272)
Supplement: Supplementary file 1 [file Table1.docx]

| **Section and Topic** | **Item #** | **Checklist item** | **Location where item is reported** |
| --- | --- | --- | --- |
| **TITLE** | | |  |
| Title | 1 | Identify the report as a systematic review. | Title page; Abstract (implied) |
| **ABSTRACT** | | |  |
| Abstract | 2 | See the PRISMA 2020 for Abstracts checklist. | Abstract section |
| **INTRODUCTION** | | |  |
| Rationale | 3 | Describe the rationale for the review in the context of existing knowledge. | \| Introduction, paragraphs 1–3 \| \| --- \| |
| Objectives | 4 | Provide an explicit statement of the objective(s) or question(s) the review addresses. | Introduction, final paragraph |
| **METHODS** | | |  |
| Eligibility criteria | 5 | Specify the inclusion and exclusion criteria for the review and how studies were grouped for the syntheses. | Research Methods section |
| Information sources | 6 | Specify all databases, registers, websites, organisations, reference lists and other sources searched or consulted to identify studies. Specify the date when each source was last searched or consulted. | Research Methods section |
| Search strategy | 7 | Present the full search strategies for all databases, registers and websites, including any filters and limits used. | Web of Science：(periostin OR POSTN) AND (allergic rhinitis) AND (biomarkers OR type 2 inflammation OR tissue remodeling)  Pubmed:  (periostin OR POSTN) AND (allergic rhinitis) AND (biomarkers OR type 2 inflammation OR tissue remodeling)  CNKI:  （主题：骨膜蛋白）OR（主题：periostin）OR（主题：POSTN）AND （主题：过敏性鼻炎） |
| Selection process | 8 | Specify the methods used to decide whether a study met the inclusion criteria of the review, including how many reviewers screened each record and each report retrieved, whether they worked independently, and if applicable, details of automation tools used in the process. | \| Research Methods section \| \| --- \| |
| Data collection process | 9 | Specify the methods used to collect data from reports, including how many reviewers collected data from each report, whether they worked independently, any processes for obtaining or confirming data from study investigators, and if applicable, details of automation tools used in the process. | A standardized form was used for data extraction. The number of reviewers and whether they worked independently were not explicitly reported; no automation tools were used. |
| Data items | 10a | List and define all outcomes for which data were sought. Specify whether all results that were compatible with each outcome domain in each study were sought (e.g. for all measures, time points, analyses), and if not, the methods used to decide which results to collect. | Research Methods section: The primary outcomes were periostin levels in different biological samples (serum, nasal secretions, tissue), correlation with clinical symptoms, diagnostic accuracy, and treatment response. |
|  | 10b | List and define all other variables for which data were sought (e.g. participant and intervention characteristics, funding sources). Describe any assumptions made about any missing or unclear information. | Research Methods section: Participant characteristics (age, gender, disease severity, comorbidities) and study characteristics (sample type, detection method) were extracted. |
| Study risk of bias assessment | 11 | Specify the methods used to assess risk of bias in the included studies, including details of the tool(s) used, how many reviewers assessed each study and whether they worked independently, and if applicable, details of automation tools used in the process. | Not performed (narrative review) |
| Effect measures | 12 | Specify for each outcome the effect measure(s) (e.g. risk ratio, mean difference) used in the synthesis or presentation of results. | \| Not applicable (qualitative synthesis) \| \| --- \| |
| Synthesis methods | 13a | Describe the processes used to decide which studies were eligible for each synthesis (e.g. tabulating the study intervention characteristics and comparing against the planned groups for each synthesis (item #5)). | Results section (thematic grouping) |
|  | 13b | Describe any methods required to prepare the data for presentation or synthesis, such as handling of missing summary statistics, or data conversions. | Not applicable |
|  | 13c | Describe any methods used to tabulate or visually display results of individual studies and syntheses. | \| Tables 1–3; Figure 2 \| \| --- \| |
|  | 13d | Describe any methods used to synthesize results and provide a rationale for the choice(s). If meta-analysis was performed, describe the model(s), method(s) to identify the presence and extent of statistical heterogeneity, and software package(s) used. | Results section (narrative synthesis) |
|  | 13e | Describe any methods used to explore possible causes of heterogeneity among study results (e.g. subgroup analysis, meta-regression). | Not applicable |
|  | 13f | Describe any sensitivity analyses conducted to assess robustness of the synthesized results. | Not applicable |
| Reporting bias assessment | 14 | Describe any methods used to assess risk of bias due to missing results in a synthesis (arising from reporting biases). | Not performed |
| Certainty assessment | 15 | Describe any methods used to assess certainty (or confidence) in the body of evidence for an outcome. | Not performed |
| **RESULTS** | | |  |
| Study selection | 16a | Describe the results of the search and selection process, from the number of records identified in the search to the number of studies included in the review, ideally using a flow diagram. | PRISMA flow diagram (Figure 1); Results section |
|  | 16b | Cite studies that might appear to meet the inclusion criteria, but which were excluded, and explain why they were excluded. | Not required |
| Study characteristics | 17 | Cite each included study and present its characteristics. | Table 1 (published periostin levels) and references cited throughout the manuscript. |
| Risk of bias in studies | 18 | Present assessments of risk of bias for each included study. | Not performed |
| Results of individual studies | 19 | For all outcomes, present, for each study: (a) summary statistics for each group (where appropriate) and (b) an effect estimate and its precision (e.g. confidence/credible interval), ideally using structured tables or plots. | Table 1; Supplementary Material |
| Results of syntheses | 20a | For each synthesis, briefly summarise the characteristics and risk of bias among contributing studies. | Results sections 2–5 |
|  | 20b | Present results of all statistical syntheses conducted. If meta-analysis was done, present for each the summary estimate and its precision (e.g. confidence/credible interval) and measures of statistical heterogeneity. If comparing groups, describe the direction of the effect. | Not applicable |
|  | 20c | Present results of all investigations of possible causes of heterogeneity among study results. | \| Not applicable \| \| --- \| |
|  | 20d | Present results of all sensitivity analyses conducted to assess the robustness of the synthesized results. | \| Not applicable \| \| --- \| |
| Reporting biases | 21 | Present assessments of risk of bias due to missing results (arising from reporting biases) for each synthesis assessed. | \| Not performed \| \| --- \| |
| Certainty of evidence | 22 | Present assessments of certainty (or confidence) in the body of evidence for each outcome assessed. | \| Not performed \| \| --- \| |
| **DISCUSSION** | | |  |
| Discussion | 23a | Provide a general interpretation of the results in the context of other evidence. | Future perspectives; Conclusion |
|  | 23b | Discuss any limitations of the evidence included in the review. | \| Section 5.3 (Standardization Challenges) and Section 5.4 (Evidence Contradictions and Interpretation) \| \| --- \| |
|  | 23c | Discuss any limitations of the review processes used. | \| Section 5.4; acknowledged as a limitation that formal risk of bias assessment was not performed. \| \| --- \| |
|  | 23d | Discuss implications of the results for practice, policy, and future research. | Future perspectives; Conclusion |
| **OTHER INFORMATION** | | |  |
| Registration and protocol | 24a | Provide registration information for the review, including register name and registration number, or state that the review was not registered. | \| The review was not registered. \| \| --- \| |
|  | 24b | Indicate where the review protocol can be accessed, or state that a protocol was not prepared. | \| A protocol was not prepared. \| \| --- \| |
|  | 24c | Describe and explain any amendments to information provided at registration or in the protocol. | Not applicable |
| Support | 25 | Describe sources of financial or non-financial support for the review, and the role of the funders or sponsors in the review. | Funding section |
| Competing interests | 26 | Declare any competing interests of review authors. | Conflict of Interest section |
| Availability of data, code and other materials | 27 | Report which of the following are publicly available and where they can be found: template data collection forms; data extracted from included studies; data used for all analyses; analytic code; any other materials used in the review. | Data Availability Statement |

*From:*  Page MJ, McKenzie JE, Bossuyt PM, Boutron I, Hoffmann TC, Mulrow CD, et al. The PRISMA 2020 statement: an updated guideline for reporting systematic reviews. BMJ 2021;372:n71. doi: 10.1136/bmj.n71. This work is licensed under CC BY 4.0. To view a copy of this license, visit <https://creativecommons.org/licenses/by/4.0/>
